# *Aeromonas* Infections in Humans—Antibiotic Resistance and Treatment Options

**DOI:** 10.3390/pathogens14111161

**Published:** 2025-11-14

**Authors:** Noelia Calvo Sánchez, Laura Sancha Domínguez, Ana Cotos Suárez, Juan Luis Muñoz Bellido

**Affiliations:** 1 Area de Microbiología, Complejo Hospitalario Universitario de Orense, 32005 Ourense, Spain; noefar@hotmail.com; 2 Servicio de Microbiología, Hospital Universitario de Salamanca, Instituto de Investigación Biomédica de Salamanca (IBSAL), Universidad de Salamanca, SACYL, CSIC, 37007 Salamanca, Spain; lsancha@saludcastillayleon.es (L.S.D.); acotos@saludcastillayleon.es (A.C.S.); 3 Departamento de Ciencias Biomédicas y del Diagnóstico, Servicio de Microbiología, Hospital Universitario de Salamanca, Instituto de Investigación Biomédica de Salamanca (IBSAL), Universidad de Salamanca, SACYL, CSIC, 37007 Salamanca, Spain

**Keywords:** *Aeromonas*, human infection, antibiotic resistance

## Abstract

The genus *Aeromonas* is widely distributed in aquatic environments, where it is a frequent fish pathogen. It has also been described in association with human infections, with most cases caused by *A. caviae*, *A. veronii* biovar *sobria*, and *A. hydrophila*. More recently, *A. dhakensis* has emerged as an increasingly important human pathogen. Transmission occurs primarily through ingestion or contacts with aquatic sources, or by consuming contaminated food, particularly from aquatic origins. Growing resistance in *Aeromonas* has been reported for penicillins (including their combinations with classical β-lactamase inhibitors), cephalosporins, and carbapenems. Among the β-lactam antibiotics, only fourth-generation cephalosporins remain almost uniformly active. Furthermore, the co-occurrence of resistance genes for third-generation cephalosporins and carbapenems within the same isolates is increasing. Recently, the presence of mobile genes conferring colistin resistance has also been documented, with resistance rates sometimes exceeding 30%. This evolution of colistin resistance is likely linked to its use in aquaculture, and together with the rise in β-lactam resistance, may be transforming *Aeromonas* into a significant reservoir of resistance genes that could potentially be transferred to species more commonly associated with human infections, such as the *Enterobacterales*.

## 1. General Characteristics of Aeromonas

The genus *Aeromonas*, belonging to the family *Aeromonadaceae*, comprises a group of Gram-negative bacilli widely distributed across numerous ecosystems, including plants and soils, but most found in aquatic environments [1]. Consisting of over 30 species, *Aeromonas* is primarily a fish pathogen [2]. However, it is also associated with infections in many other animals (insects, reptiles, amphibians, and birds [3]), as well as in humans. The genus has gained increasing importance in human medicine, particularly due to the emergence of antimicrobial resistance, such as to colistin [4].

Physiologically, *Aeromonas* is classified into two main groups [5]:

- Psychrophilic, non-motile *Aeromonas*: This group, designated *Aeromonas salmonicida*, has an optimal growth temperature of 22–25 °C and infects reptiles and fish.
- Motile, mesophilic aeromonads: This group, with an optimal growth temperature of 35–37 °C [5,6], includes most species commonly isolated as human pathogens [3].

**Methodology**. We conducted a search of the last 10 years in PubMed and Embase using the following criteria: ((“Aeromonas”[MeSH Terms] OR Aeromonas[tiab] OR “A. hydrophila”[tiab] OR “A. caviae”[tiab] OR “A. veronii”[tiab])
AND
(humans[MeSH Terms] OR human*[tiab])
AND
(infection*[tiab]

From the results obtained, reviews, meta-analyses, and studies providing quantitative or percentage data on the incidence of antimicrobial resistance were selected, as were those providing information on resistance mechanisms, their distribution and prevalence, and new therapeutic proposals.

## 2. *Aeromonas* as a Human Pathogen

Of the more than 30 species described in this genus so far, nineteen are considered emerging human pathogens. Recent studies suggest that over 95% of reported human cases are caused by three species—*Aeromonas caviae*, *Aeromonas veronii* biovar *sobria*, and *Aeromonas hydrophila* [1]—and recently joined by *Aeromonas dhakensis*, which is mainly linked to wound infections [7]. The first description of its role in human disease did not occur until 1954 [8].

Human infections with *Aeromonas* are primary associated with gastroenteritis, though its etiological role is often unclear. It is also linked to wound infections, skin and soft tissue infections, and, in some cases, bacteraemia and sepsis [9]. Classically, it has been considered a much less frequent pathogen than major gastrointestinal pathogens like *Salmonella*, *Shigella*, *Campylobacter*, and *Escherichia coli* [10] or skin and soft tissue pathogens such as *Staphylococcus aureus* and *Streptococcus pyogenes* [11]. However, it is likely an under-diagnosed pathogen, especially when its identification is based on biochemical activity panels [12].

A 2006 study from France on 78 *Aeromonas* isolates [13] showed that 44% of infections involved wounds, 26% were bacteraemia, 19% were enteritis, 6% were respiratory infections, and 5% were other infections. Overall, 40% of the isolates were *A. veronii*, 35.7% were *A. hydrophila*, and 21.4% were *A. caviae*. Notably, *A. hydrophila* predominated in wound infections, *A. caviae* in bacteraemia, and *A. veronii* had a more even distribution across the different infection types.

Studies conducted mainly in Asian and African countries estimate the prevalence of *Aeromonas* isolates in diarrheal patients to be less than 5% of total diarrheal samples [14,15,16,17,18]. Regarding species and subspecies frequency, *A. caviae* and *A. veronii* rank well above *A. hydrophila* as enteric pathogens in virtually all studies [19].

A recent study from Australia [20] also showed a clear predominance of *A. veronii* bv. *sobria* in fecal samples (66.7% of *Aeromonas* fecal isolates), while *A. dhakensis* predominated in wound samples, followed by *A. hydrophila*. In blood cultures, the most frequent species were *A. dhakensis* and *A. caviae*, which together accounted for more than 63% of blood cultures positive for *Aeromonas* spp. A more recent Australian study, focusing only on fecal isolates, also identified *A. veronii* as the most frequent species causing gastreoenteritis (52%), followed by *A. caviae* (27%) and *A. hydrophila* (12.5%) [21].

A study conducted in Japan on samples that were predominantly extraintestinal (97.4%) reported *A. caviae* (60%) as the most common species, well above *A. hydrophila* (17%), *A. veronii* (14%), and *A. dhakensis* (6%) [22]

The main prevalence results are shown in Table 1.

However, species differentiation in different studies may be biased by both geographical factors and the method used for identification. Identification performed by MALDI-TOF MS and sequencing of the *rpoB* and *gyrB* genes allows for reliable identification of species like *A. dhakensis*, whose identification by classical biochemical methods is much more complex and less reliable. Similar results in purulent infections, with a clear predominance of *A. dhakensis*, have also been published by other authors [28]. This behavior is likely associated with the higher virulence of *A. dhakensis* compared to other *Aeromonas* species, such as *A. veronii*, *A. caviae*, and *A. hydrophila*, as demonstrated in the fibroblast C2C12 cell line, BALB/c mouse, and *Caenorhabditis elegans* models [23,29]. Furthermore, a type 6 secretion system (T6SS), has been shown in animal models to be associated with an increased propensity for producing disseminated infections [30]. Exotoxin A has been shown in several studies to be an important virulence factor for *Aeromonas*, demonstrating high virulence in both *C. elegans* [28] and mouse models [24].

A recent study suggests that patient age may also determine which species are most prevalent [31]. In this study, *A. veronii* was the most prevalent species across all ages, but especially so in young adults. *A. caviae* was more frequent in young children and adults over 60, as were *A. hydrophila* and *A. dakhensis*.

Similarly, a recently published study from Iran suggests that *Aeromonas* infection may be more prevalent in immunocompromised individuals. This study, conducted on 130 immunocompromised patients with diarrhea (mostly hematological patients), showed that the prevalence of *Aeromonas* was significantly higher than that of *Clostridioides difficile*, and up to 10 times higher than that of *Campylobacter jejuni* [32].

Although the presence of specific virulence genes has been demonstrated in the SSU strain, comparative studies of the genomes of different *A. dhakensis* strains show that the presence of these genes is not constant. While there is relevant evidence for higher virulence of *A. dakhensis* [28], the specific virulence mechanisms remain insufficiently defined.

*Aeromonas* infections are preferentially associated with certain risk factors (e.g., advanced age or immunosuppression) [31], but can occur in patients with no known risk factors and across any age group [32]. This is due to their ability to overcome host immune mechanisms, which allows them to infect both immunosuppressed and immunocompetent patients [8]. Their pathogenesis is multifactorial and is associated with the wide number of enzymes and toxins they can produce, such as proteases, lipases, hemolysins, cytotoxins, and enterotoxins [33].

A recent study [34] not only demonstrated that the predominant species differ between intestinal infections (*A. veronii*) and extraintestinal infections (*A. caviae* and *A. hydrophila*), but also that certain genes are more frequent in intestinal isolates. These include *act* (encoding an enterotoxin), *aexT* (encoding an ADP ribosyltransferase toxin involved in the cytotoxicity of *A. salmonicida*), and *asc*F-G (encoding a type III secretion system). In contrast, pathogenicity factors like *alt* (encoding a thermolabile *lip* cytotoxic enterotoxin), *hly*A (encoding a hemolysin), and *ela* (encoding an elastase), were significantly more frequent in isolates from extraintestinal infections.

Transmission primarily occurs through ingestion or contact with aquatic environments, which constitute its primary habitats [35]. However, its resistance to standard water purification procedures may represent an alternative source of human transmission [36]. Another source of transmission is the consumption of contaminated plant or animal food, especially aquatic products that are not cooked [30].

One factor that may be important in modulating the pathogenicity of *Aeromonas* is the temperature of its aquatic habitat, which is particularly relevant in the context of global warming. In several *Aeromonas* species, the warming of their habitat above usual temperatures has been shown to increase the expression of various virulence factors [2,37]. Indeed, numerous enzymes and toxins have been identified in *Aeromonas* that can act as pathogenicity factors, such as the capsule itself, type 2, 3, and 6 secretion systems, DNAases, proteases, lipases, elastases, adhesins, hemolysins, cytotoxins, and enterotoxins [38,39,40,41,42,43,44].

## 3. *Aeromonas*: Antibiotic Resistance

As is the case with other Gram-negative bacilli, *Aeromonas* is a genus capable of developing resistance to numerous antibiotics [4].

Most *Aeromonas* species can exhibit antimicrobial resistance, regardless of the origin of the isolates. In *Aeromonas*, the production of inducible cephalosporinases is common, and the presence of plasmid-mediated carbapenemases was described as early as the 2000s [45]. Different species of *Aeromonas* (*A. hydrophila*, *A. caviae*, *A. veronii*, and *Aeromonas schubertii*) have shown their ability to produce β-lactamases of classes B, C, and D [46]. The presence of TEM group ESBLs, specifically TEM-24, has occasionally been described in a clinical isolate, likely acquired from an *Enterobacter aerogenes* clone that was highly prevalent in France at that time [47].

A 2006 study from France on 78 *Aeromonas* isolates [13], showed low levels of susceptibility to amoxicillin, and first-generation cephalosporins in *A. hydrophila* as well as in *A. veronii* and *A. caviae*. Susceptibility rates were much higher for third- and fourth-generation cephalosporins, carbapenems, fluoroquinolones, and aminoglycosides (Table 2).

In recent years, clinical isolates resistant to third-generation cephalosporins and carbapenems have been described in numerous countries [48,49,50]. In studies from the 1990s [51,52], CphA was the most common carbapenemase in *Aeromonas*. It is an inducible chromosomal metallo-β-lactamase found in many *Aeromonas* species. However, since 2008, the presence of typically plasmid-encoded carbapenemases, such as VIM, IMP [44,48], and NDM [49,53], began to be reported. Data from major antibiotic resistance studies are shown in Table 2.

Currently, genes encoding both classical β-lactamases and metallo-β-lactamases as well as other genes associated with antibiotic resistance, are frequently found in most *Aeromonas* species, with no major differences between clinical and environmental isolates [41,42,43]. This expansion of plasmid-mediated carbapenemases is occurring in clinical settings worldwide, and is most likely related to the global increase in carbapenem use due to the growing spread of ESBL, mainly in enterobacteria.

Studies of environmental isolates suggest high levels of resistance in non-human ecosystems. This points to a possible environmental reservoir of resistance genes, which poses a clear danger for the transfer of these multidrug-resistant (MDR) profiles to microorganisms, both of this genus and others, linked to human infections.

Previous studies have demonstrated the ability of *Aeromonas* to acquire and donate DNA through transformation [54], similarly to other microorganisms such as *Streptococcus pneumoniae*, at least under laboratory conditions.

Regardless of their ability to acquire DNA from other microorganisms through transformation, a probably decisive element in the spread of antimicrobial resistance in *Aeromonas* is the acquisition of resistance genes via mobile genetic elements. The existence of integrons has been demonstrated in numerous *Aeromonas* species, including some of the most frequent human pathogens. Associated with these integrons are different cassette genes linked to resistance to various antimicrobial families (e.g., trimethoprim, chloramphenicol, β-lactams, and aminoglycosides) [55].

Several studies have demonstrated the possibility of transferring different genetic elements associated with antimicrobial resistance between human digestive pathogens and environmental *Aeromonas*. Thus, *Aeromonas* can acquire these resistance elements and, at the same time, act as a source for species that may eventually become pathogens [56,57,58].

A recent meta-analysis shows that resistance levels in clinical isolates of *Aeromonas* are generally not alarming when compared to current levels of other Gram-negatives, such as enterobacteria, *Pseudomonas*, or *Acinetobacter*. Resistance levels to gentamicin are 10.8%, to third- and fourth-generation cephalosporins they are between 7.7 and 12.6%, and to ciprofloxacin and levofloxacin they are 7–8% [59].

A very recent study focused only on human *Aeromonas* infections diagnosed over the last 14 years [25], covering 112 isolates, also shows acceptable susceptibility levels to aminoglycosides, third-generation cephalosporins, aztreonam, and fluoroquinolones, with resistance rates below 7.1% in all cases. However, resistance percentages for carbapenems are in the 40–60% range, which suggests a significant spread of either CphA or other carbapenemases described in *Aeromonas* in the first decade of the 2000s.

These results align with a 2015 study from Spain, which found imipenem resistance figures in clinical isolates of 34.5%, much higher than those found in environmental sources (8.8%) and in fish fauna (18.8%) [60].

In contrast, a study from China published in 2019, based on isolates obtained between 2015 and 2017, shows slightly higher resistance percentages for some third-generation cephalosporins (ceftriaxone, 14.8%), but still below 10% for fourth-generation cephalosporins (cefepime, 4.3%), aminoglycosides (gentamicin, 5.2%), and fluoroquinolones (ciprofloxacin, 6.1%) [16]. Surprisingly, and in contrast to what had already been reported in other studies at that time, carbapenem resistance percentages were also below this figure (imipenem, 2.6%) [16].

Another interesting finding in this study [16] is the clear difference in resistance levels observed in isolates from intestinal vs. extraintestinal infections. For example, resistance was significantly higher in extraintestional isolates for imipenem (25.9% vs. 2%), ceftriaxone (70.6 vs. 5.1%), cefepime (23.5% vs. 1%), gentamicin (23.5% vs. 2%), and ciprofloxacin (35.3% vs. 1%).

Another study from China published in 2023 [38], on 188 intestinal and extraintestinal isolates of *Aeromonas* obtained between 2013 and 2020, also shows significantly higher resistance levels in extraintestinal isolates for ceftazidime (22.8% vs. 6.7%), ceftriaxone (34.8 vs. 6.7%), imipenem (23.3% vs. 3.2%), and ciprofloxacin (20.2 vs. 3.3%). It is possible that these differences are also related to the different species distribution, since in this study, more than 50% of the intestinal isolates corresponded to *A. veronii*, while *A. caviae* and *A. hydrophila* predominated in the extraintestinal isolates.

The study of resistance mechanisms in these strains shows that CphA remains the most frequent carbapenemase. However, the co-presence of different types of carbapenemases with conventional TEM and ESBL β-lactamases, mainly from the CTX-M group, in some isolates is concerning, and may pose significant therapeutic challenges [38].

In this regard, it should be noted that a recently published article on clinical isolates from Iran between 2018 and 2020 reported the presence of ESBLs in 60% of isolates, with a clear predominance of CTX-M, and the presence of VIM, KPC, NDM, and IMP-type carbapenemases in 50%, regardless of the predictably high presence of CphA, which was not collected in the study [49].

However, a study conducted in China specifically on invasive *Aeromonas* infections in hematological patients does not confirm these high levels of resistance in invasive infections, except for carbapenems. In general, resistance percentages for the most common antibiotics remain below 10% (ceftazidime, 6.1%; ceftriaxone, 8.2%; cefepime, 0%; aztreonam, 8.2%; levofloxacin, 2.1%; and gentamicin, 4.1%). Only carbapenems show significantly higher figures (imipenem, 70.8% and meropenem, 71.4%) [61].

Recent studies focused on *A. dhakensis*, a species whose clinical importance has grown rapidly, also show similar data: relatively low resistance figures for third- and fourth-generation cephalosporins, aztreonam, and aminoglycosides, with high resistance figures for carbapenems, ranging from 41.9% for meropenem to 76.9% for imipenem [62].

More recently, *A. caviae* subsp. *aquatica*, a new multiresistant *Aeromonas* subspecies isolated from drinking water reservoirs, has been described in Brazil. The isolate shows a worrying resistance profile, being resistant to ampicillin, ceftazidime, cefepime, aztreonam, carbapenems, and fluoroquinolones. Genomic analysis shows that it produces different beta-lactamases, such as bla_MOX-6_ and bla_OXA-427_/bla_OXA-504_; mutations in parC; other antibiotic-resistance genes, such as *qnr*A; and multidrug efflux pumps, along with several genes encoding virulence factors [63].

A study of *Aeromonas* obtained from hospital wastewater showed that 97% carried *cphA* and 39.4% carried *bla*KPC. It was also shown that *bla*KPC was located on plasmid susceptible to transfer to other species [64], suggesting that in some particularly critical areas, such as the hospital environment, the presence of these resistance mechanisms may be growing rapidly.

A recent study in Iraq on nosocomial isolates shows a prevalence of carbapenem resistance in *A. sobria* of 58%. Of these isolates, most (57.1%) were blaGES carriers, followed by blaKPC (28.6%) [65].

Another study recently published in China, in a pediatric population, on 13,662 samples obtained from children with diarrhea between 2016 and 2023, shows that the prevalence of *Aeromonas* was relatively low (only 2.5%, compared to almost 16% of *Salmonella* cases). *Aeromonas* was more frequent in the group of children between 6 and 11 months of age and during the summer months, and the most prevalent species were *A. caviae* (53.3%) and *A. hydrophila* (22.6%). Antibiotic resistance was high for ampicillin (40–42%) and ampicillin/sulbactam (35–40%), ranging between 5 and 11% for fourth-generation cephalosporins, and was between 0 and 1% for fluoroquinolones and carbapenems [26].

Some studies recently published in Latin America, such as the one published in 2025 in Argentina on 37 samples of human origin, from both intestinal and extraintestinal locations, show a fairly similar prevalence for *A. hydrophila* (37.8%), *A. veronii* (32.4%), and *A. caviae* (29.7%). As in studies from other geographical areas, resistance was high or very high to first-generation cephalosporins (67.6%), ampicillin, and ampicillin/sulbactam (94.6%), but low to cefotaxime (8.1%), cefepime, piperacillin/tazobactam, fluoroquinolones, and aminoglycosides (2.7%). However, resistance to imipenem and meropenem was high (35–42%) [27]. Moreover, in Latin America, and more specifically in Brazil, the emergence of some worrying strains has recently been reported, such as the *A. veronii* TR112 strain, which simultaneously produces several transferable genes resistant to beta-lactams (blaVEB28, blaCphA3, and bla OXA912) and colistin (mcr-3 and mcr-3.6) [66]. An isolate with worrying multidrug resistance characteristics was also recently described in China, although it was not a clinical isolate but rather an isolate obtained from wastewater. In this case, it was an isolate of A. caviae carrying two mrc alleles (mrc-3.43 and mrc-7.2) and two carbapenemases (blaNDM-1 and blaKPC-2) simultaneously [67].

With regard to studies available in Europe, a study published in Germany in 2024 on 55 human isolates, both from gastroenteritis and other extraintestinal infections, shows a clear predominance of *A. veronii* biovar *sobria* (32.7%) and *A. caviae* (30.9%) over *A. hydrophila* (16.4%), *A. salmonicida* (3.6%), and *A. dhakensis* (0.8%), although this study does not provide data on antibiotic sensitivity [25].

A study recently published in the United States on 105 *Aeromonas* isolates, both extraintestinal (mostly) and intestinal, shows that approximately 50% of cases were caused by *A. hydrophila*, with a prevalence much higher than *A. caviae*, which causes 16% of cases; *A. sobria*, which causes 11%; and *A. veronii,* which accounts for less than 3%. As in other studies, the figures for insensitivity are very high for penicillins and combinations of amoxicillin/clavulanic acid and ampicillin/sulbactam, remaining around 40–50% for carbapenems, and around or below 10% for third- and fourth-generation cephalosporins, fluoroquinolones, and aminoglycosides [25].

In recent years, articles have been published on specific anatomical locations of *A. hydrophila* infection. A study published in 2025 on osteoarticular infections caused by *A. hydrophila* recorded between 2020 and 2024 reports 58 infections, mostly linked to mechanical crushing or lacerations. Thirty-one percent were monobacterial infections, while in the rest, *A. hydrophila* was involved along with other Gram-positive and Gram-negative bacteria. In this case, the resistance rates were higher, with resistance to ampicillin, amoxicillin/clavulanic acid, and cefazolin exceeding 80%, to gentamicin and tetracyclines around 40%, to fluoroquinolones between 15% and 25%, and to meropenem around 20%. In contrast, resistance to cefotaxime and cefepime was less than 10% [68].

Overall, the comparability of these results is limited, given the number of factors that may influence resistance levels (e.g., human or non-human origin, intestinal or extraintestinal origin of the isolates, and the species involved). It appears that intestinal and extraintestinal isolates mostly correspond to different species, and this likely has a significant influence on the resistance figures found. Similarly, the fact that some studies were conducted in immunocompromised patients may mean that the species associated with extraintestinal cases are different, thus modifying the resistance figures. This is without forgetting that the greater or lesser use of different antimicrobial families, both at the clinical level and in other areas, can impact the overall resistance figures for both *Aeromonas* and other bacterial genera.

## 4. Colistin Resistance

A cause for concern regarding *Aeromonas* and antimicrobial resistance is colistin resistance. The previously cited meta-analysis [59] shows an overall colistin resistance of 21.2%, which rises to 30.7% in clinical strains.

Colistin is a cationic peptide antibiotic mainly used for severe infections caused by MDR Gram-negative bacteria. Colistin was obtained from *Bacillus polymyxa* in 1947 and licensed for intravenous therapeutic use in the 1950s [69]. Due to its considerable toxicity and side effects, colistin has always been an antimicrobial with restricted use, which has likely contributed to its high levels of susceptibility over the years.

The main target for colistin is the outer cell membrane of Gram-negative bacteria. The cationic colistin binds to the anionic lipid A, an essential component of the LPS of Gram-negative bacteria. This interaction displaces Ca^2+^ and Mg^2+^ ions from the LPS, disrupting the permeability barrier of the outer membrane. This, in turn, increases the uptake of polymyxin, leading to the loss of cellular contents and ultimately bacterial killing [70,71,72,73]. Resistance to colistin can arise trough different mechanisms, such as capsule formation, overexpression of efflux pumps [72], or loss of LPS through mutations in the *lpx*A, *lpx*C, and *lpx*D genes [71]. However, resistance primarily occurs due to a series of modifications in the LPS structure, that reduce its negative charge, thereby decreasing its affinity for colistin [74,75,76].

Despite colistin being widely used in livestock farming, for both therapeutic and prophylactic purposes [77], colistin resistance remained infrequent until recently [78,79]. It was commonly believed that resistance was primarily mediated by chromosomal mutations, which limited its capacity for horizontal transfer [79]. These chromosomal processes involved chromosomal mutations (e.g., *pmrA/pmrB*, *phoP/phoQ*, and the *mgrB* gene) that trigger the *arn*BCADTEF operon, as well as the phosphoethanolamine (pEtN) transferase gene *pmrC* (also known as *eptA*), which facilitates the synthesis and transfer of aminoarabinose (L-Ara4N) and pEtN to lipid A [80], modifying its charge and reducing its affinity for colistin [81].

A decisive factor in the dissemination of colistin resistance among Gram-negative bacteria has been the horizontal transfer of plasmid-borne genes, such as mobile colistin resistance (*mcr*). MCR belongs to the pEtN enzyme family, and its expression results in the incorporation of pEtN into lipid A, leading to lipid A charge modification and a decrease in colistin affinity [77,82,83,84].

In 2015, the first plasmid-mediated mobile colistin-resistance gene (*mcr-1*) was discovered in *E. coli* isolated from pigs in China [85]. Subsequently, *Enterobacterales* carrying *mcr-1* have been detected worldwide. Additional *mcr* determinants, from *mcr-2* to *mcr-10*, along with several variants, have been reported [86,87,88,89,90,91,92,93].

It is believed that *mcr* genes originated from intrinsic, chromosomal *mcr*-like genes present in *Moraxella*, *Aeromonas*, *Shewanella*, and other mostly environmental bacteria [94].

Colistin resistance in *Aeromonas* has been described in various areas, including aquaculture, though it is not the most widely used antimicrobial in this field, as its use is much lower than that of other antimicrobials like tetracyclines, quinolones, amoxicillin, macrolides, sulfonamides, and amphenicols [95]. Resistance to colistin seems to be more frequent in the clinical setting [96]. A 2016–2017 study conducted on 6497 bacterial isolates from 13 provinces in China demonstrates the presence of *mcr*-3 in 49 of them, of which eight corresponded to different species of the genus *Aeromonas* [97]. The presence of *mcr* genes varies greatly from one country to another, partly because colistin has never been marketed or approved for non-human use in some countries. However, the importation of food from areas where this restriction does not exist may change this situation, as some very recent studies have shown [98].

In a recent 2022 study of 144 clinical *Aeromonas* isolates of from six tertiary hospitals in Japan, the most frequent species were *A caviae* (87 isolates), *A hydrophila* (25 isolates), *A veronii* (20 isolates), and *A dhakensis* (9 isolates). This study showed the presence of *mcr*-3 genes in 28% of the isolates (mainly in *A. hydrophila* and *A. dakhensis*), and *mcr*-7 in 13% (mainly in *A. veronii*) [99].

The emergence and worldwide dissemination of *mcr* has endangered colistin’s efficacy, raising public health concerns [22].

## 5. Treatment Alternatives

As early as 1995, it was suggested that some of the most empirically used antimicrobials for systemic infections, such as penicillins and their combinations with classical β-lactamase inhibitors (amoxicillin/clavulanic acid and piperacillin/tazobactam) were not considered adequate options for the treatment of *Aeromonas* infections. This was because the activity of these penicillins was irregular and β-lactamase inhibitors did not substantially improve their efficacy [100]. However, at that time, the use of third-generation cephalosporins and carbapenems was still advocated, due to the low prevalence of resistance.

The study by Sakurai et al. [22] shows high resistance levels for piperacillin/tazobactam (30%), third-generation cephalosporins (34% for cefotaxime), and carbapenems (24.3% for imipenem). This susceptibility profile means that the failure of conventional empirical treatments cannot be ruled out. In fact, an analysis of 105 patients with *Aeromonas* hepatobiliary infection included in this study, shows that only 46% received appropriate antimicrobial therapy within 48 h of diagnosis.

A study published in Korea in 2016 [101], on 242 cases of *Aeromonas* bacteraemia registered between 2000 and 2013, reported lower resistance percentages, around 15% for piperacillin/tazobactam, 10–15% for third-generation cephalosporins, and 10% for carbapenems. Despite this, the percentage of initial empirical treatment considered inadequate was over 40%.

A recently published study from Saudi Arabia [89] on 24 cases of human *Aeromonas* infections recorded between 2015 and 2022, showed a resistance rate of 58.3% for piperacillin/tazobactam, 75% for ceftriaxone, 83.3% for ceftazidime, and 62.5% for meropenem. These figures are even higher than those from previous studies [102]. However, fourth-generation cephalosporins (cefepime), fluoroquinolones, and aminoglycosides still perform very well, showing no resistance among the isolates tested.

It is difficult to arrive at a complete overview of the treatment for human *Aeromonas* infections, as the number of reported cases with specific treatments is scarce, and most are individual case descriptions. However, given the sensitivity data reported in recent studies [88,89,90,91,92], it seems prudent, especially in severe invasive cases and when risk factors suggest the possibility of *Aeromonas* infection, to avoid using penicillins, including combinations with classical β-lactamase inhibitors, third-generation cephalosporins, and carbapenems. Instead, resorting to fourth-generation cephalosporins, aminoglycosides, and fluoroquinolones is preferable.

However, the available resistance data, especially in extraintestinal infections, make it necessary to consider new alternatives. In this sense, the new β-lactams and the new β-lactam–β-lactamase inhibitor combinations that have emerged in recent years may represent an important contribution, particularly in extraintestinal infections, due to their severity and the greater frequency of resistant isolates.

The new fifth-generation cephalosporins (ceftaroline and ceftobiprole) do not provide significant improvements as their activity against Gram-negative infections is generally similar to that of third-generation cephalosporins [103]. With regard to new β-lactam–β-lactamase inhibitor combinations, avibactam, vaborbactam, and relebactam inhibit class A β-lactamases (including KPC) and class C β-lactamases. Avibactam and relebactam also partially inhibit class D β-lactamases, allowing for good coverage of Gram-negative bacilli, except for metallo-β-lactamase producers.

For isolates producing class B β-lactamases, the combination of ceftazidime/avibactam with aztreonam and cefiderocol may be an alternative, although experience is limited. However, it should be noted that, in isolates producing this type of carbapenemases, other antimicrobials such as colistin, aminoglycosides, and fluroquinolones are often active, though they are frequently associated with less bactericidal activity or more toxicity.

In any case, the rapid spread of resistance to β-lactam antibiotics, including carbapenems, along with the spread of plasmid-mediated colistin resistance, poses a dual risk. First, human *Aeromonas* infections will have increasingly complex treatments. Second, *Aeromonas* may become a reservoir of resistance genes, potentially leading to increases in resistance to antimicrobials that are not yet a widespread problem, such as colistin. To prevent this, the prudent use of antimicrobials is needed in human therapeutics, as well as a thorough control of their use in other areas such as animal nutrition and aquaculture.

Phage therapy and immunotherapy represent promising therapeutic strategies for combating bacterial infections, though in the case of *Aeromonas* research has primarily focused on their application in aquaculture rather than human disease [104,105,106,107,108]. However, a recent study [109] investigated the protective effects of a novel myovirus phage, vB_AceP_Pac, and concluded that it holds “promising potential” for treating diarrhea caused by *A. caviae*.

Another compelling application of phages is their potential to mitigate the prevalence of antibiotic resistance. For instance, a recent study [110] suggested that a combination of two *A. hydrophila* phages (vB_AhaP_PZL-Ah8 and vB_AhaP_PZL-Ah1) could enhance therapeutic efficacy both in vitro and in vivo. The frequency of resistance mutations was found to be significantly lower in *A. hydrophila* cells treated with the phage mixture compared to those treated with a single phage (*p* < 0.01).

While these findings are not immediately translatable to human infections, they contribute valuable knowledge that may become increasingly relevant as the problem of antibiotic resistance in human pathogens continues to grow.

In few cases will the importance of the “One Health” concept be as evident as in this one.

## Figures and Tables

**Table 1:** *Aeromonas* species prevalence in different studies (%).

Ref.	Prevalence	*A. hydrophila*	*A. caviae*	*A. sobria*	*A. veronii*	*A. dhakensis*	Origin of Isolates
[13]	3.1	9	34	26	29	#	Enteric
[14]	4.25	2.7	1.6	#	#	#	Enteric + extraintestinal
[15]	4.3	5.7	25.3	#	42.5	#	Enteric and environment
[16]	*	5.2	41.7	#	31.3	13.9	Enteric + extraintestinal
[17]	*	1.0	86.7	#	12.2	#	Enteric
[18]	*	20	14	#	21	39	Enteric + extraintestinal
[19]	*	17.1	14.5	#	18.4	48.7	Wound
[23]	*	3.5	68.1	#	15.5	#	Enteric and environment
[24]	*	26.1	28.7	#	25	18.1	Enteric + extraintestinal
[25] **	*	65.7	31.4	11.4	2.9	#	Enteric + extraintestinal
[26]	2.4	26.6	53.3 ##	1.5 ***	#	#	Enteric
[27]	*	37.8	29.7	#	32.4	#	Extraintestinal

* No overall prevalence specified in the study; # not studied; ** 17/105 of patients had mixed infections by *A. hydrophila*/*A. caviae* or *A. hydrophila*/*A. jandaei*; ## *A. punctata*; *** *A. jandaei*.

**Table 2:** Antibiotic susceptibility (% susceptible+%intermediate) of *Aeromonas* in different studies.

Reference	AMP/AMOX	A/C/A/S	CIP/LEV	SXT	CRO/CTX	CFP	GEN/AK	IMP/MER
[13]	5.6	100	94.5	91.7	100	*	100	100
[13]	14	*	54	60	76	100	100	100
[15]	14	*	98	99.8	*	*	*	*
[16]	6.1	87	93.9	94.8	85.2	95.7	94.8	97.4
[19]	*	*	100	*	97.3	100	100	89.2
[29]	1.2	6.2	75.8	83.9	97.3	97.6	93.7	76
[24]	*	*	95.7	*	70.2	89.4	*	95.2
[25]	*	74	96.3	90.6	93.6	99	97.9	63
[26]	59	63.2	97.3	99.4	*	91.9	*	99.1
[27]	5.4	5.4	97.3	89.2	91.9	97.3	97.3	64.9

AMP: Ampicillin; AMOX Amoxicillin; A/C: Amoxicillin/clavulanic acid; A/S: Ampicillin/sulbactam; CIP: Ciprofloxacin; LEV: Levofloxacin; SXT: Trimethoprim + sulphamethoxazole; CRO: ceftriaxone; CTX: Cefotaxime; CFP: Cefepime/Cefpirome; GEN: Gentamicin; AK: Amikacin; IMP: Imipenem; MER: Meropenem. * Not studied.

## Data Availability

Not applicable.
